# Overall Accessibility to Traveling by Rail for the Elderly with and without Functional Limitations: The Whole-Trip Perspective

**DOI:** 10.3390/ijerph111212938

**Published:** 2014-12-12

**Authors:** Catherine Sundling, Birgitta Berglund, Mats E. Nilsson, Ragne Emardson, Leslie R. Pendrill

**Affiliations:** 1 Gösta Ekman Laboratory, Department of Psychology, Stockholm University, SE-106-91 Stockholm, Sweden; 2 Institute of Environmental Medicine, Karolinska Institutet, P.O. Box 210, SE-171-77 Stockholm, Sweden; E-Mail: birgitta.berglund@ki.se; 3 Department of Psychology, Stockholm University, SE-106-91 Stockholm, Sweden; 4 Gösta Ekman Laboratory, Department of Psychology, Stockholm University, SE-106-91 Stockholm, Sweden; E-Mail: mats.nilsson@psychology.su.se; 5 SP Technical Research Institute of Sweden, P.O. Box 857, SE-501-15 Borås, Sweden; E-Mails: ragne.emardson@.sp.se (R.E.); leslie.pendrill@sp.se (L.R.P.)

**Keywords:** accessibility, travel behavior, functional limitation, barrier, railway travel, older persons

## Abstract

Elderly persons’ perceived accessibility to railway traveling depends on their functional limitations/diseases, their functional abilities and their travel behaviors in interaction with the barriers encountered during whole trips. A survey was conducted on a random sample of 1000 city residents (65–85 years old; 57% response rate). The travels were perceived least accessible by respondents with severely reduced functional ability and by those with more than one functional limitation/disease (e.g., restricted mobility and chronic pain). Those who traveled “often”, perceived the accessibility to be better than those who traveled less frequently. For travelers with high functional ability, the main barriers to more frequent traveling were travel costs and low punctuality. For those with low functional ability, one’s own health was reported to be the main barrier. Our results clarify the links among existing functional limitations/functional abilities, the barriers encountered, the travel behavior, and the overall accessibility to traveling. By operationalizing the whole-trip concept as a chain of events, we deliver practical knowledge on vulnerable groups for decision-making to improve the transport environment for all.

## 1. Introduction

As the proportion of *the elderly* increases in the world, transport systems must be improved to meet the requirements of this potentially vulnerable group. In the year 2060, almost 25% of the Swedish population is expected to be over 65 years old, as compared to 19% in 2011. The situation is similar in many Western countries [1]. As people are expected to live longer, and also preserve an active lifestyle longer, the total number of journeys made by the elderly will increase. Concomitantly, the number of persons with functional limitations will increase with increasing age and, therefore, many elderly can be expected to acquire more than one functional limitation. So far, our current knowledge is limited regarding older persons’ travel behavior and specific travel needs. However, as every fourth person will be 65 years or older, the Swedish transport system will be challenged continuously to provide services, particularly if not developed sustainably in time to meet the special needs for this age group [2].

Research has shown that mobility, including accessible public transport, contributes to a better quality of life for many elderly persons. However, the group of “elderly” constitutes a heterogeneous group. As regards age, for instance, persons aged 65–74 have been found to spend (voluntarily) an additional 70 min a day outside the home compared to persons aged 85 or more, implying that increasing age is negatively associated with mobility [3]. Accessible public transport may help older persons to stay mobile longer in life and is important when visiting family and friends, for shopping and for physical exercise and sport events, among other activities [4]. To be able to reach desired people and places, highly valued characteristics are comfortable modes of transportation and easiness getting on and off vehicles. Conversely, uncomfortable and cramped vehicles and complicated routes could create pain in persons with movement and joint problems, for example, if they must walk to distant bus stops or get on and off busses without drop-steps.

Many elderly persons would like to engage in out-of-home activities more often, but transportation deficiencies constitute a main obstacle [5,6,7]. Schmöcker* et al.* [8] reported that use of public transport for shopping purposes was negatively associated with existing functional limitation. For travelers with cognitive deficits, serial tasks and high complexity of the travel environment may be especially demanding [9]. Low priced tickets have been shown to be a particularly important facilitator for older persons’ use of public transport [10]. Bus stop density has showed to encourage elder persons to travel by bus more frequently but the same result has not been found for rail and underground station density [8]. Examples of other facilitators for the elderly and for persons with functional limitations are short walking distances at stations and reliability of service. However, features in the surrounding environment may be equally important. For example, dropped curbs at pavements on the way to and from stations/stops, are important for wheelchair users. The whole trip has to be “seamless” from start to finish [11].

### Definition of Concepts

**Figure 1:**
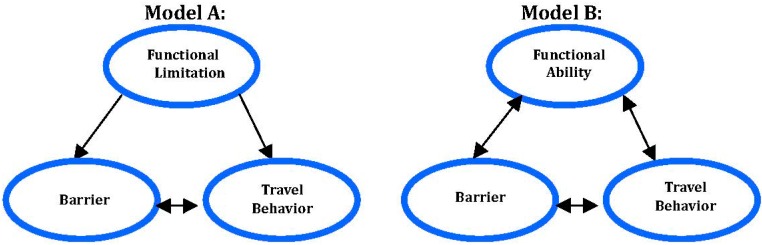
Two overall accessibility models: Model A is for travelers without or with various kinds of functional limitations (and diseases) and Model B is for the same travelers’ degrees of functional ability. In the models, functional limitation (and disease) is invariant, relative to travel behavior and the barriers, whereas functional ability is variant because of reciprocal interactions.

To what extent do elderly persons’ perceptions of overall accessibility in public railway transport depend on earlier traveling experiences? Apart from the travelers’ own functional limitations and their level of functional ability when confronting specific barriers during whole trips, travel behavior will be critical to their future traveling (see Figure 1). Moreover, would the sort of functional limitation or the level of functional ability influence or even create the kind of barriers a person will encounter, and/or affect his/her travel behavior? A further question is if the specific barriers encountered would be associated with travel behavior. An overall goal is to be able to improve the accessibility to travel for groups of travelers, who are often neglected in transport research, that is, persons with various sorts of functional limitations.

No single definition of *accessibility* seems to be generally accepted. Gould [12] described the concept as a slippery notion, one of those common terms that everyone uses until faced with the problem of defining and measuring it. The general key distinctions though, in the most recent definitions, are that accessibility is a joint attribute of people and places [13]. Notably, we are focusing here on passengers’ perceptions of accessibility. Earlier research has shown that public-transport suppliers tend to overestimate their service quality, if compared with customers’ perceptions and evaluations of it [14,15]. Please note that the term *railway accessibility* here refers to the respondent’s perceived access to traveling, by train only. Additionally, the term *overall accessibility* is here regarded as a more complex and general construct (Figure 1: Model A and Model B), referring to any kind of traveling, not only railway. It is composed of persons’ *functional ability* (their *kind*s of *functional limitations* and other personal features, inclusive), the *barriers* they would encounter in the transport system, and their self-reported *travel behaviors*. Moreover, we have adopted *the whole trip concept*; the trip is regarded as a chain of events, a *travel chain*, including everything from planning the trip to arriving at the intended destination (cf. [16,17,18]).

A *functional limitation* is here defined as a reduction in inherently existing physical, psychological/behavioral or intellectual abilities (e.g., restricted mobility, vision or cognitive impairments). A functional limitation is thus a characteristic of a person as such. In the present research context, it should be separated from functional ability or disability, which is *not* a personal characteristic. The concept of disability has been internationally agreed upon to be “the product of an interaction between a set of personal features and various characteristics of the environment”; see e.g., WHO [19]. Similarly, *functional ability* is here defined as the *perceived level* of functioning involved in person-environment interaction [20].

We define *barriers* as constraints in the *travel chain,* experienced by the individual passenger (cf. the whole-trip concept adopted above). Even during the same trip, travelers will experience partly the same and partly different kinds of barriers. Over time, barriers will vary also for the same individual, for example because of short-term changes in a functional limitation such as a sprained ankle. Concomitantly, the level of functional ability will also change. If a barrier has already been encountered during a trip, it might alter personal experiences and attitudes to traveling, and may thus affect, adversely, the perception of additional barriers and, again, functional ability. Or, conversely, having encountered a barrier may in the future help a person to overcome it. A longitudinal study design would be required in order to detect such sequential dependencies.

If barriers are reduced or removed, the traveler’s functional limitation is still present and invariant, but his/her functional ability may improve. As a consequence, the resulting travel behaviors might be more flexible and independent. Therefore, our research aims at determining overall (and railway) accessibility to traveling as a function of functional ability, barriers encountered, and travel behaviors of persons with and without functional limitations. In spite of all the efforts to reduce barriers, especially for those with functional limitations, we still do not know what actions are most efficient for improving the (functional) ability to travel and to establish independent travel behaviors.

We define *travel behavior* as the way people use different modes of transport; our focus is mainly on the frequency of traveling and the travel mode. In Sweden and in many other countries [4,21,22], research has shown that, in the age group 65–84 years old, the car is the most frequently used travel mode. However, for this age group, car usage declines with increasing age of users, just as mobility does, in general. Measured in movements per day, car driving stands for 38% and being the passenger for 15%. There are differences though, within this age group (65–84 years old), related to income and gender; for instance, persons with higher income and the men more often possess a driver’s license and a car. In contrast, for this age group of older persons, only 0.7% of the journeys are by long-distance train, or only 2%, if trips with the underground and tram are included. In Sweden and internationally, the most common reason for older persons to travel is shopping; physical exercise and recreational activities are also common [4,23].

Travel experiences and their meaning may differ among individuals. For persons with functional limitations, the different limitations may lead to various kinds of problems in the travel environment. Moreover, the severity of the same functional limitation as well as other factors, such as earlier experiences, would affect the experience and interpretation of the travel situation, and thus the person’s perceived ability to travel. Similarly, older persons do not constitute a homogenous group [22]. Therefore, it is necessary to develop ways of measuring accessibility from the perspective of the individual travelers, with various prerequisites in the transport system. Presently, there is a shortage of measuring instruments adapted for these individual users with varying functional abilities, as grounded in their unique functional limitations.

## 2. Research Problems

Our six main research problems concern *whole-trip traveling* involving *trains* (short and long distances), restricted to *elderly* persons with and without functional limitations, all living in the County of Stockholm.

- To model *overall accessibility* grounded in elderly travelers’ functional ability, their travel behavior and the barriers encountered in the environment.
- To explore how *railway accessibility* is related to functional ability, travel behavior and barriers in the travel environment for the elderly.
- To determine *functional ability* and its relationships to kinds and number of *functional limitations/diseases* in the elderly.
- To determine *barriers* encountered in the whole-trip travel environment by the elderly with varying degrees of *functional ability* and kinds of *functional limitations/diseases*.
- To determine *travel behavior* in the whole-trip travel environment as a function of *functional ability* for older persons with various kinds of *functional limitations/diseases*.
- To determine *travel behavior* as a function of *barriers* encountered by the elderly with varying *functional ability* and kinds of *functional limitations/diseases*.

## 3. Method

### 3.1. Theoretical Model

In Figure 1, we propose a triangular model for conceptualizing *overall accessibility* to the whole trip, railway accessibility inclusive, or for applying the model to railway accessibility only. In the model, accessibility arises from travelers’ functional limitations (and/or their functional abilities), the barriers encountered in the transport system, and traveling behavior. Model A shows that functional limitations may influence what barriers are encountered in the travel environment and, therefore, also the travel behavior of the individual. Please note that a *functional limitation* is here regarded as a person factor, inherent in the individual, and thus, in the short term, it is “invariant” in relation to the environment, e.g., a broken leg or reading/writing disabilities are the same regardless of the situation. In contrast to Model A, Model B builds on three pairwise and reciprocal interactions. Here, the “invariant” construct functional limitation has been replaced by the “variant” construct *functional ability* (or* disability*).

We are in this research focusing on the traveler’s ability rather than her or his disability [24]. Model B shows that barriers encountered in traveling will interact with and influence a person’s functional ability as well as travel behavior. Each of these three constructs constitutes complex, multi-variable quantities (or patterns) among which interaction outcomes are of paramount importance for measuring the quantity *accessibility,* railway accessibility inclusive. By reducing the barriers, the ability of traveling will be improved, and thus bring about more flexible travel behaviors. Barriers are here regarded as specific to each trip, and they will emerge from the person–environment interaction in the travel chain. In Model A as well as in Model B, the interaction among barriers encountered in traveling and travel behavior are reciprocal.

In the present research, we have developed a questionnaire that is a prototypical measuring-instrument built on our accessibility models (Figure 1). An overall goal is to pinpoint the most effective accessibility improvements in the transport system for persons with functional limitations and reduced functional ability. So far, a few instruments have been developed for measuring public-transport accessibility for persons with functional limitations. Two of them are the “travel chain enabler” restricted to bus transport [17,25] and the accessibility measure for the weighting of barriers in public transport by Emardson* et al.* [26].

### 3.2. The Sample and Procedure

The present study was conducted in accordance with the Declaration of Helsinki. After approval from the local ethical committee in the Stockholm area (project identification code 2011/1169-31/5), a pilot study was conducted in May–June 2011 in order to try out and improve our specifically developed questionnaire that was to be used in the main study. Our piloting was based on a convenience sample of 22 persons (64–76 years old); all questionnaires were returned. The content and design of the questionnaire were improved according to these results. The main improvements were on the layout, clarity of language, and on decreased number of response alternatives; all to make it easier for respondents to complete the questionnaire. More space was also made available for respondents’ potential comments.

In November 2011, a random sample of 1000 persons (65–85 years old) living in the County of Stockholm, Sweden (population approx. 2,000,000) was taken. The questionnaire was mailed to each respondent’s home address together with a reply-paid envelope. An introductory letter was attached, which contained information regarding the goal and content of the investigation as well as the voluntary participation.

Three reminders were sent out. The last reminder also contained a copy of the questionnaire. Three months later, the data collection was closed. By then, 574 questionnaires had been filled in and returned; the overall response rate was 57%. The questionnaire data were coded and analyzed statistically in SPSS version 20.0 [27].

### 3.3. The Self-Report Questionnaire

Our questionnaire used basic demographic questions from recent Swedish surveys, as well as a few questions on feelings of insecurity in the transport system. The authors developed a larger part of the questionnaire. The questionnaire was especially built for* whole trip* traveling and the questions were developed accordingly; overall accessibility was the overarching construct. It is here regarded as the outcome of four constructs: functional limitation/disease, functional ability, barriers, and travel behavior (see Figure 1). Railway accessibility is introduced as a fifth construct, constituting a partial construct of overall accessibility, which in turn refers to the whole trip. We focus on the interrelationships among these five constructs as well as on their relationships to our sixth overarching construct; *overall accessibility*. For definitions of all our constructs, please see Section 3.4 below.

*Demographic questions* inform that out of the 574 respondents, 54% were women and 46% men. Thus, the gender split of respondents agrees with the gender distribution in Stockholm County [28]. The arithmetic mean age of our respondents was 73 (Md = 72) years, the standard deviation 5.8 years, and the range 65–85 years. Table 1 shows that most respondents were retired (in all 95%): 32% reported living alone (the same proportion applies for the country [29]), 58% with a spouse, and 8% with a spouse and child/children. Considerably reduced functional ability was more common among the women than the men, and among the older respondents (≥75–85 years old) as compared to the younger ones (65–74 years old). This result is in line with previous research [30,31,32]. Those with low income had to a higher extent reduced functional ability. This outcome is on par with earlier enquiries [33]. Notably, five of our respondents with mobility service reported that they had no reduction at all in their functional ability. A plausible reason might be relapsing illnesses. We chose to use Cramér’s V as a statistical effect-size measure of association among variables. It is commonly used for data in larger than two by two matrices, and it takes into account the degrees of freedom.

**Table 1:** Demographic characteristics of random sample of elderly respondents in Stockholm County.

Descriptive		Reduction in Functional Ability			
	All *n* (%)	None *n* (%)	Somewhat *n* (%)	Very *n* (%)	Cramér’s V (*p*)
Gender:					0.11 (0.026)
Women	312 (54)	177 (58)	109 (36)	21 (7)	
Men	262 (46)	141 (56)	106 (42)	6 (2)	
Age:					0.14 (0.004)
65–74	314 (55)	192 (63)	104 (34)	10 (3)	
75–85	257 (45)	124 (49)	110 (44)	17 (7)	
Retired:	568 (95)	295 (56)	206 (39)	26 (5)	0.06 (0.321)
Income/month (SEK):					0.17 (0.000)
0–16 499	148 (27)	62 (43)	71 (49)	12 (8)	
16 500–33 499	228 (42)	121 (54)	89 (40)	12 (5)	
33 500 and above	165 (30)	116 (71)	47 (29)	1 (1)	
Living:					0.09 (0.107)
Family/nursing home	390 (68)	229 (60)	135 (35)	17 (4)	
Alone	181 (32)	89 (51)	77 (44)	10 (6)	
Car in household:	408 (71)	240 (60)	148 (37)	11 (3)	0.17 (˂0.001)
Driver’s license:	458 (80)	262 (58)	174 (39)	13 (3)	0.18 (˂0.001
Discount card:	439 (77)	266 (62)	158 (37)	6 (1)	0.23 (˂0.001)
Mobility service:	43 (8)	5 (12)	22 (52)	15 (36)	0.33 (˂0.001)

Note: The total sample *n* = 574, range of age: 65–85 years old. The symbol n stands for subsamples of respondents. Only the loadings with absolute values greater than 0.3 are shown.

### 3.4. Six Constructs and Their Measurement

The questionnaire was focused on the barriers encountered in the whole-trip traveling environment, especially railway traveling, including travelers’ past experiences and their expectations for future traveling. Another important part of the questionnaire investigated the travelers’ functional limitations/diseases*,* their own (perceived) functional ability, their use of assistive devices (e.g., a cane or glasses), and Special Transport Service (*i.e.*, Swedish Färdtjänst). The questionnaire also researched actual travel behavior. Taken together, our set of six constructs constitutes the measurement model of* overall accessibility.* One question addressed accessibility in train traveling specifically, here denoted as railway accessibility. Our six constructs are measured as follows:

- *Railway accessibility:* The perceived railway accessibility was measured in one question (5-category scale).
- *Functional limitation/disease:* Self-reported functional limitations/diseases were assessed by one question with 15 response categories. The categories were selected through literature review of functional limitations and of transport research. Here, functional limitation/disease refers to a medical diagnosis or symptom. Three additional questions assessed authority-evaluated needs, e.g., mobility service, disabled parking permit and one question on disability aids (eight response categories).
- *Functional ability:* Self-reported degree of functional ability and health status (5-category scales). Functional ability refers to the self-reported *severity* of one’s functional limitation(s) or disease(s).
- *Travel behavior:* Actual travel behavior was assessed with questions on the following eight travel aspects: travel frequency, mode of conveyance, destination, purpose of trip, ticket purchase, luggage brought, travel companion(s), and change of transportation modes.
- *Barrier:* The main part of the questionnaire measured the perceptions of specific barriers in the travel environment encountered during *the whole trip* (*i.e.*, the whole travel chain, door-to-door). Subsections were on barriers in: (a) long-distance train traveling, (b) train traveling in general, and (c) public transport, including other transport modes than train. The questions on barriers were in the format of scales and open questions. Additionally, one question contained 30 alternatives regarding willingness to travel more often by long-distance train depending on different potential barriers. Thus, most barriers were pre-defined by us and selected through literature review, although the respondent were also allowed to suggest barriers.
- *Overall accessibility:* Taken together, the five empirical constructs presented above [(a) railway accessibility; (b) functional limitation/disease; (c) functional ability; (d) travel behavior; and (e) barriers)], constitute the theoretical construct “overall accessibility” to whole-trip traveling.

In assessing functional limitation/disease and functional ability, it may be argued that authority-evaluated needs would be more “objective”. However, in the present research we attach great importance to *self-reports*, because the respondents’ decisions regarding their travel behavior and earlier experiences in the travel environment would depend on their own appraisal of their functioning. Thus, two persons with the same functional limitation may experience the travel environment differently, and consequently also their needs. Therefore, they may draw different conclusions leading to different behaviors.

We focused on a densely populated geographic living area, the County of Stockholm, Sweden. As most travel environments, the Greater City of Stockholm is invariably changing depending on season, construction work, larger events,* etc*. The questionnaires were sent out in November 2011 and most of them were returned in November and December. The data collection closed on 1 February 2012. During the data collection, a large rebuilding project at the Stockholm Central Station was going on. This might have further influenced, for instance, the ability to move around inside the station and affected information retrieval and orientation somewhat.

## 4. Results of Questionnaire Study

This section presents the results for five constructs, together constituting the concept of overall accessibility (see Figure 1): functional limitations/diseases, functional ability, barriers, travel behavior, and railway accessibility. First, we will report on each of these constructs separately, followed by descriptions of how functional limitations influence the other constructs and how these latter are interrelated.

### 4.1. Functional Limitations/Diseases

The functional limitations/diseases most commonly reported by the elderly were: vision impairment (22%), hearing impairment (21%) and cardiovascular disease (17%). Consequently, the most frequently used assistive aids were glasses or lenses (76%). The coefficients of rank-order correlation between pairs the 15 kinds of functional limitations/diseases were generally low; most Spearman’s ρ-values were below 0.30; the three highest statistically significant coefficients were, for *p* = 0.01:

(a). cognitive deficits in attention/memory/concentration *and* reading/writing/speech (ρ = 0.40, *n* = 7),
(b). musculoskeletal impairments with chronic pain *and* restricted mobility (ρ = 0.39, *n* = 31), and
(c). sensory impairments regarding hearing *and* vision (ρ = 0.32, *n* = 56).

As expected, the younger portion of our participants (below 75 years) had fewer concurrent *functional limitations/diseases* (see Table 2 for the 15 of the functional limitations/diseases as response categories) than the older portion (75 years and above). A larger part of the less old participants reported no or only one functional limitation/disease, whereas our older participants more frequently reported two or more (significantly different for *p* = 0.005 in multiple regression). No significant difference in the number of concurrent functional limitations/diseases was found for gender.

**Table 2:** Reduction in functional ability as a function of the kind of functional limitation/disease (FL/D) for the study sample of elderly; the 15 kinds of FL/D listed in order of decreasing value of Cramér’s V.

Functional Limitation/Disease ^1^	Degree of Reduction in Functional Ability ^2^			N ^1^	Cramér’s V ^3^ Value (*p*)
	None (a) *n* (%)	Somewhat (b), (c) *n* (%)	Very (d), (e) *n* (%)		
No functional limitation/disease	106 (80)	19 (18)	2 (2)	127	0.23 (˂0.001)
Restricted mobility	7 (9)	56 (69)	18 (22)	81	0.46 (˂0.001)
Chronic pain	8 (14)	35 (64)	12 (22)	55	0.34 (˂0.001)
Reading, writing or speech disability	0 (0)	6 (50)	6 (50)	12	0.32 (˂0.001)
Attention, memory or concentration disability Chest disease	4 (17) 4 (16)	13 (56) 16 (64)	6 (26) 5 (20)	23 25	0.24 (˂0.001) 0.21 (˂0.001)
Rheumatic disease	5 (19)	16 (62)	5 (19)	26	0.20 (˂0.001)
Cardiovascular disease	34 (35)	56 (58)	6 (6)	96	0.20 (˂0.001)
Hearing impairment	47 (40)	63 (53)	9 (8)	119	0.18 (˂0.001)
Vision impairment	49 (40)	62 (51)	10 (8)	121	0.17 (˂0.001)
Mental ill-health	4 (24)	11 (65)	2 (12)	17	0.12 (0.017)
Neurological disorder	3 (25)	8 (67)	1 (8)	12	0.09 (0.086)
Diabetes	29 (48)	26 (43)	6 (19)	61	0.09 (0.095)
Asthma, allergy, hyper- sensitivity	29 (45)	31 (48)	4 (6)	64	0.08 (0.164)
Epilepsy	2 (40)	2 (40)	1 (20)	5	0.07 (0.270)
Travel sickness Respondent suggested FL/D	11 (58)	8 (42)	0 (0)	19 67	0.04 (0.600)

Notes: ^1^ N stands for number of persons reporting the different kinds of functional limitations/diseases as well as the group of persons reporting none. In total, 736 reports of functional limitations/diseases were given for the 15 kinds of functional limitations/diseases; in addition, 127 respondents (out of 551) reported no functional limitation/disease. The total sample of questionnaire respondents was 574; ^2^ Response categories for functional ability: (a) not reduced; (b) somewhat reduced; (c) reduced; (d) very reduced; (e) extremely reduced. The symbol n stands for subsamples of respondents in each degree of reduction in functional ability; ^3^ Cramér’s V is based on the three here presented degrees of functional ability (out of 5).

#### 4.1.1. Age and Gender

All coefficients of correlations were low between the age of the participant and the presence of each of the functional limitation/disease (15 kinds). Here, as well as elsewhere, we have used the Pearson’s coefficient (*r*) whenever measures were given on interval or ratio scales (e.g., age). The highest coefficients of correlation for age were found for restricted mobility (*r* = 0.22, *n* = 81), attention/memory/concentration (*r* = 0.18, *n* = 23) and reading/writing/speech (*r* = 0.12, *n* = 12). These correlations were all statistically significant for *p* = 0.01.

#### 4.1.2. Jointly Existing Functional Limitations/Diseases

A principal components analysis (PCA) was conducted on the correlation matrix of the prevalence of the 15 functional limitations/diseases (in total *n* = 745, for each of the 15, *n* = 5–121; see Table 3). The aim of this analysis is to find out if there are underlying patterns of association among the various functional limitations/diseases of the subjects. Table 3 shows the seven components of the PCA, extracted for eigenvalues >1. Together, these principal components explain 60% of the total variance. There are two main sets of components: C1–C3 that refer to the functional limitations and C4–C7 that refer to the diseases. Among the *functional limitations*, the first principal component (C1) is dominated by “cognitive, language (reading, writing and speech) and mental symptoms, mental ill-health included”, the second component (C2) constitutes “chronic pain and restricted mobility, neurologically associated conditions inclusive”, and the third component (C3) consists of the two main sensory impairments: “hearing and vision”; also “diabetes” may contribute because of visual deficits. Among the *diseases*, the fourth component (C4) consists of “cardiovascular and lung-associated diseases”, the fifth component (C5) of “neurological disorders, epilepsy inclusive”, the sixth component (C6) of “systemic diseases (rheumatic disease and diabetes)” and, finally, the seventh component (C7) of travel sickness alone.

**Table 3:** Principal components analysis (PCA) of functional limitations/diseases for the study sample of the elderly.

Functional Limitations/Diseases	PCA Component ^1^
*Functional Limitations:*	
Attention, memory or concentration disability	C1 (0.83)
Reading, writing or speech disability	C1 (0.72)
Mental ill-health	C1 (0.54)
Chronic pain	C2 (0.79)
Restricted mobility	C2 (0.74)
Hearing impairment	C3 (0.78)
Vision impairment	C3 (0.73)
*Diseases:*	
Chest disease	C4 (0.68)
Cardiovascular disease	C4 (0.60)
Asthma, allergy, hypersensitivity	C4 (0.59), C5 (41)
Epilepsy	C5 (0.80)
Neurological disorder	C2 (0.34), C5 (0.51)
Rheumatic disease	C6 (0.77)
Diabetes	C3 (0.40), C6 (0.55)
Travel sickness	C7 (0.55)

^1^ C1-C7 refer to the 7 extracted components in a PCA of the coefficients of correlation (*r*) between pairs of functional limitations/diseases. The loadings of the seven components are given within parenthesis (in total, 60% of the variance is explained).

*Mobility service and parking permit.* According to our questionnaire study, 8% of the respondents had by the authorities been declared to be in need of Special Transport Service (Sw. “Färdtjänst”), a taxi service for persons with functional limitation/disease. This is a low percentage for our sample of older persons (age 65–85 years), because as many as 4% of the whole Swedish population receive such service [34]. In our sample, 3% had a disabled parking permit.

### 4.2. Functional Ability

In our random sample (age group of 65–85), 67% reported that, if compared to others of the same age, their general health was “good or very good” (on a 5-point category scale from “very good” to “very poor”). This stands comparison with the 63% in Statistics Sweden’s survey for the age group of 65–84 years [33]. Similarly, most of our respondents (57%) reported having “no reduction” in their *functional ability* (on a 5-point scale from “not reduced” to “extremely reduced”). There was no significant gender difference. Only 5% of our respondents reported that their functional ability was “very reduced” or “extremely reduced”. These large reductions were found to be more common among the women than the men.

### 4.3. Barriers

The questionnaire mainly focused on train traveling, including the underground, local trains (including trams), commuter trains, and long-distance trains. However, it also included questions on public transport in general, because different modes of transport are often involved in the same travel. One section of the questionnaire addressed specifically long-distance train journeys. Notably, the whole journey can be affected critically by the encounter of only one barrier in the whole travel chain. In public transport, the underground was the travel mode most frequently avoided, especially during evenings (16%) and nights (24%). The most important barriers for feeling secure were poor information on departure times (65%) and unpleasant driving behavior (61%).

*Traveling by train in general.* What kind of barriers had the elderly *train-traveling* respondents encountered? In our open questions, the most frequently reported barriers were: (a) delays and other time-matters of train travel; (b) shortcomings in the physical environment; and (c) shortcomings in the information available. The most frequently suggested improvements to the train-traveling experience concerned time aspects. These included wishes for “more frequent departures” and “better punctuality”, for example, through “improved snow clearance”. Not only time-saving journeys were called for, conversely, some respondents wanted “more time available at the stations”, such that they would not need to rush when boarding.

*Physical travel-environment.* In open questions, many respondents proposed how to improve the physical environment onboard the train and/or in station areas. More personal space onboard, such as seats for all passengers or more space between seats, pleasant temperature in trains and waiting rooms, as well as more escalators, are examples of wished improvements of the physical travel environment. Many wishes concerned improved service at stations and onboard, e.g., more staff available to help out with luggage and/or food and drink, especially better food quality, more to choose from, or table service, because balancing food and drink in full speed trains can be a challenge.

*The weather.* The weather was found to affect 30% of the respondents (“often” or “sometimes”, measured on a 4-point scale) in deciding whether they would travel by train or not, although not being affected by the weather at all was equally common (32%). Notably, those who would like to travel by train more often were slightly more affected by the weather (41%) than the other respondents.

**Figure 2:**
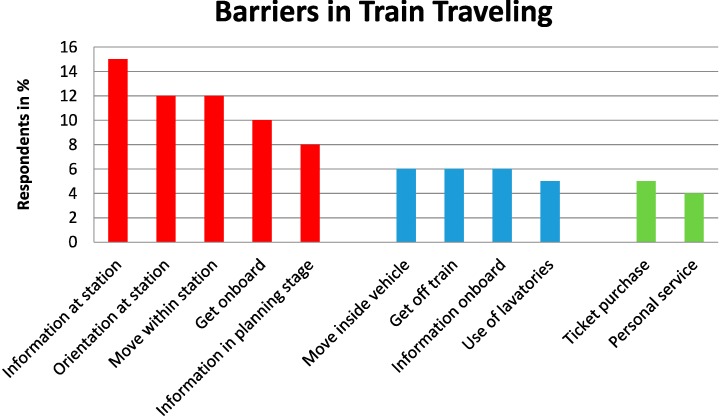
Whole-trip traveling: Barriers in long-distance train traveling reported by the respondents who (*n* = 145) “had traveled by long-distance train last year”.

*Long-distance travel chain (the whole trip).* One section of the questionnaire was devoted to barriers perceived during different parts of the travel chain of the most recent long-distance train journey; that is, from the planning of the trip to reaching the destination. The participants were asked how easy or difficult it was (on a 5-point scale) to use the travel environment, and they could also contribute with comments. Here, we present the results in order of importance according to the respondents. Thus, the parts of the travel chain that most of the respondents found problematic are presented first. Overall, among those who had traveled in the last year, a majority found it easy to travel. Figure 2 shows the distribution of barriers they had encountered in the travel environment. The most frequently reported barrier was “the inability to obtain information at the stations”: 15% found it “relatively difficult” or “very difficult” to retrieve such information. “More staff” and “improved visual information” were requested. Main complaints were about “inconsistent information or signs that were out of order”.

Because of inadequate information, ongoing reconstruction work at stations and long distances within station areas, 12% of the respondents found orientation and movement within station areas difficult. Moreover, 10% experienced difficulties getting onboard the train, and the main concern was to be able to climb high steps into the train with luggage. Better information during the planning stage of the journey was wished for (8%), e.g., because of difficulties in understanding the ticket system. Moving around inside the vehicle was perceived to be difficult because of insufficient stability in the moving train, because of vibrations, jerkiness,* etc*. When getting off the train, again the high steps constituted a barrier to carrying luggage, but also because of nonexistent or unhelpful staff. Regarding information onboard the train, loudspeakers were the main cause of discontent; the auditory message being too hard to hear, too loud or the acoustic system simply out of order. Some respondents had experienced inconveniences because of lavatories that were closed, dirty, or out of function or simply lumbered with luggage. A “long wait for ticket purchase” or “too few choices for seat reservations” or “kinds of credit cards accepted” are all examples of complaints from those who found ticket purchase difficult (5%). Moreover, during the journey only 4% found the personal service from the staff unsatisfactory, however, mainly because of absence of staff.

*Ticket price and punctuality.* To get a lower ticket price was the most important factor for choosing to travel more often by long-distance trains. If it would have been *less expensive*, 62% said they would have traveled more often, followed by the factor *punctuality* (keeping the schedule and arriving at destination on time). Other factors were somewhat less important, such as the possibility to travel at other hours, the other passengers’ attitudes or not having to travel underground. Appendix shows a complete list of the 30 response alternatives that were presented to the respondents as well as the percentages of responses. A PCA on the 30 barrier alternatives did not deliver any useful n-dimensional patterns for improving the travel environment.

### 4.4. Travel Behavior

On average, our elderly respondents traveled 23 days per month, measured as sum of days per month traveled with each mode of transport. (Notably, 29% of the respondents missed this question). The travel modes involved long-distance and short-distance trains, buses, airplane, boat, and car, taxi inclusive, as well as Special Transport Service. Respondents scaled travel frequency on a 5-point category scale (end points: “never” to “4–7 days per week”). For each kind of transport, we have calculated *travel frequency* as “the sum of the number of days per month traveled”. The respondents were excluded if they had omitted one or more of the sub-questions on travel modes. The results for the travel modes were:

(a) *Train traveling.* The respondents were found to travel on average 7 days per month with train. Our concept “train traveling” includes underground, commuter trains, trams, local trains, and long-distance trains.
(b) *Car use.* For the group of respondents as a whole, the car was the most frequently used travel mode: 93% traveled by car at least occasionally and 36% used it 4–7 days per week. A majority of the respondents had both driver’s license and a car in the household (see Table 1). Of those who responded to both these questions (driver’s license *and* car ownership), 92% of the men and 71% of the women, owned a driver’s license, but 78% of the men and only 49% of the women had a car in the household. Thus, fewer women than men could independently use a car in the household. Although most respondents traveled by car as driver or passenger, at least occasionally, a majority of our respondents (77%) also used public transport often enough to have acquired a discount card.
(c) *Local bus and the underground.* Apart from the car, local bus was the most frequently used modes of travel, followed by the underground.
(d) *Long-distance bus and aircraft*. Long-distance bus was the least used modes of transport, indeed 76% of the respondents reported *never* using it. Most of the respondents (66%) also traveled by air, occasionally.
(e) *Long-distance train*. More than half of our respondents, 61%, never traveled by long-distance train. Moreover, most of our respondents (all 65–85 years old) had *not recently* traveled by long-distance train. Therefore, the part of the questionnaire concerned with the long-distance train journeys during the last year only involved 145 persons (or 25% of all respondents). Our respondents with no car in the household or with high income were more likely to have traveled by train during the last year than those with a car or with lower income (odds ratios were 3.5 and 2.1, respectively).
(f) *Traveling abroad*. Most of the respondents traveled abroad, at least once in a while. Only 13% reported that they never traveled outside Sweden. But, during the last year, a majority of the respondents (86%) *had not* made a long-distance journey or journey abroad. Of those who *had*, more than half had traveled “by car” (60%), followed “by air” (48%) and/or “by train” (28%). The respondents were also asked what transport mode they would normally use for travels abroad or long-distance journeys; and “by air” was the most common answer (81%).
(g) *Buying tickets.* Ticket purchases were most commonly made at a staffed point-of-sale (83%), followed by the Internet (59%) and by telephone service (42%). Ticket-machine (26%) and cell-phone (10%) purchases were least common. The percentage of missing answers was high for these questions, that is, up to 43%. Still, it is worth noticing that in our group of elderly, staffed point-of-sale was the most preferred.

### 4.5. Railway Accessibility

A majority of the respondents (59%) considered the accessibility to train traveling as being “very good” or “fairly good”. Conversely, 10% thought it was “very bad” or “rather bad”. According to 31%, it was in between. Among all respondents, 41% would have liked to travel by train more often if it would have been possible. This view was more common among those without (45%) than those with (34%) functional limitations, but this difference is not statistically significant.

In the following sections, railway accessibility is researched empirically by scrutinizing the kinds of interrelationships (unidirectional and bidirectional) among pairs of the different constructs presented in Models A and B (see Figure 1 above). The difference between the two *models of accessibility* is basically that, in Model A, accessibility to whole-trip traveling is viewed as grounded in a static feature of the person (his/her functional limitation), whereas, in Model B, accessibility is viewed as originating in the reciprocal interactions between pairs of all three constructs, that is, the person’s degree of functional ability, the barriers met and the travel behavior potentially accomplished. In both models, there is an interaction between the barriers and travel behavior. Notably, Model A only contains this one reciprocal relationship, the one between travel behavior and barriers. In Model B, functional ability is an interactive factor in relation to both barriers and travel behavior.

### 4.6. Interrelationships among Four Basic Research Constructs

In this section, we present relationships among our targeted constructs for the respondents *with* or *without* different *kinds* of functional limitations/diseases (Model A) as well as for the respondents with different *degrees* of functional ability (Model B). Apart from the three constructs involved: the barrier(s) encountered in traveling, the resulting travel behavior and existing functional limitation(s) *or* the functional ability, we now, additionally, introduce the overarching construct of railway accessibility in traveling. For the set of constructs involved in Model A or Model B, please consult Figure 1.

#### 4.6.1. Model A—Barriers Identified for Travelers’ Kinds of Functional Limitations/Diseases

Regarding the barriers identified in different parts of the latest journey with *long-distance train*, the most commonly reported barrier was “inadequate information at stations”. However, the dominant kinds of barriers naturally varied among the travelers because of their different kinds of functional limitation/disease. Thus, persons with neurological diseases or with restricted mobility found it particularly difficult “to move around onboard the long-distance train” (*r* = 0.46, *n* = 2 or *r* = 0.31, *n* = 14, respectively). Together with rheumatic diseases (*n* = 4), neurological diseases (*n* = 2) additionally implied “moving problems within the stations” (both *r* = 0.32). Because of the limited number of respondents of each kind of limitation/disease, these between-group comparisons should be interpreted cautiously. Regarding potential barriers for traveling more often with long-distance train, only weak associations were established for the different kinds of functional limitations/diseases (see Appendix for the complete list of 30 potential barriers and Table 2 and Table 3 for functional limitations/diseases).

#### 4.6.2. Model A—Travel Behavior Identified for Travelers’ Kinds of Functional Limitations/Diseases

Travel frequency was generally weakly correlated (Pearson’s r) with the kinds of functional limitation/disease (in total *n* = 398). Having restricted mobility or problems with attention/memory/concentration were negatively correlated with travel frequency (*r* = −0.14 or *r* = −0.12, respectively, both significant at the *p* = 0.01 level). Similarly, vision impairment correlated reversely, but significantly with travel frequency (*r* = −0.13 at the *p* = 0.05 level). Davidsson [35] reports that persons with restricted mobility and vision impairment tend to travel less frequently.

#### 4.6.3. Model A—Railway Accessibility for Travelers’ Kinds of Functional Limitations/Diseases

The railway accessibility (category) scale co-varied to the greatest extent with restricted mobility (Spearman ρ = 0.14, *n* = 74) or chronic pain (ρ = 0.12, *n* = 51) than was the case with other functional limitations/diseases. Although low, these rank-order coefficients of correlation are significant at the *p* = 0.01 level (see Table 4). For those with mental ill-health (ρ = 0.10, *n* = 17) and epilepsy (ρ = 0.09, *n* = 5), the coefficients of correlation were significant at the *p* = 0.05 level. No significant coefficients of correlations were obtained between railway accessibility and the remaining 11 kinds of functional limitations/diseases listed in Table 4.

**Table 4:** Spearman’s coefficients of correlation (ρ) between railway accessibility and each of the 15 functional limitations/diseases reported by subsamples (*n*).

Functional Limitation/Disease	ρ	*n*
Travel sickness	−0.04	16
Restricted mobility	0.14 **	74
Vision impairment	0.04	115
Hearing impairment	0.05	113
Reading, writing or speech disability	0.07	11
Attention, memory or concentration disability	0.07	20
Chronic pain	0.12 **	51
Asthma, allergy, hypersensitivity	0.04	60
Mental ill-health	0.10 *	17
Cardiovascular disease	0.04	90
Chest disease	0.07	23
Epilepsy	0.09 *	5
Neurological disorder	0.04	12
Rheumatic disorder	−0.01	24
Diabetes	−0.02	57

** (ρ) is significant at the 0.01 level (2-tailed); * (ρ) is significant at the 0.05 level (2-tailed).

#### 4.6.4. Model B *vs.* Model A: Functional Ability for Person’s with Various Kinds of Functional Limitations/Diseases

In comparing functional ability with each of the different kinds of functional limitations/diseases, it is important to note that functional ability is a continuous variable, whereas functional limitation/disease constitutes a set of classes. As shown in Table 2, some functional limitations or diseases were more often than others reported to cause serious reductions in functional ability (in Table 2, the functional limitations/diseases are listed in the order of decreasing value of Cramér’s *V*). Respondents with the functional limitations/diseases, restricted mobility or chronic pain, reported that they had reduced functional ability to a higher extent (Cramér’s *V* = 0.46 and 0.34, respectively, *p* < 0.001) than, for example, the respondents with impaired vision or hearing (Cramér’s *V* = 0.17 and 0.18, *p* < 0.001). In comparing the reduction in functional ability for the different kinds of functional limitations (Table 2), the 5-category response scale of ability has been collapsed to a 3-category scale (none, somewhat and very). The reason for this was to increase the group size for each response category and thus to create a better basis for statistical testing, (see Table 1, Table 2 and Table 4 for the compiled data: “none” = “not reduced”; “somewhat” = “somewhat reduced” & “reduced”, and “very” = “very reduced” & “extremely reduced”).

Table 5 shows the relation between functional ability and the respondents’ number of functional limitations/diseases. Respondents with “two” or more functional limitations/diseases reported their *functional ability* to be lower than those with “no” or only “one” functional limitation/disease (Cramér’s *V* = 0.429, *p* < 0.001). Thus, among the participants who had “no” or only “one” functional limitation/disease (65%), a majority reported that they had “no reduction” in their functional ability (column 1 of Table 5), whereas most of the respondents with” two” or more functional limitations (35%) reported that they had “somewhat” or “very reduced” functional ability (Cramér’s *V* = 0.43). (Please note that the perceived ability level was measured in one question only). We here chose to focus on *perceived* functional ability as it is a direct measure. Notably, some of the participants “without functional limitation/disease” responded that they had “a reduced functional ability”. This was logically possible because of the fixed number of response alternatives for the functional limitations/diseases in the questionnaire. Respondents may thus have suffered from other kinds of health problems.

**Table 5:** Reduction in functional ability as a function of number of different functional limitations/diseases per respondent (*n* = 555, Cramér’s *V*^ 1^ = 0.429, *p* < 0.001).

Number of Functional Limitations/Diseases Per Respondent ^2^	Degree of Reduction in Functional Ability ^3^			
0	**None (a) *n* (%)**	**Somewhat (b), (c) *n* (%)**	**Very (d), (e) *n* (%)**	***n***
0	149 (81)	33 (18)	2 (1)	184 ^4^
1	111 (62)	64 (36)	4 (2)	179
2	38 (38)	58 (58)	4 (4)	100
3	12 (24)	31 (63)	6 (12)	49
4	2 (10)	16 (80)	2 (10)	20
5	1 (7)	10 (67)	4 (27)	15
6–8	0 (0)	3 (60)	5 (62)	8

Notes: ^1^ Cramér’s V is based on the three here presented degrees of functional ability (out of 5) for the 15 listed functional limitations/diseases; ^2^ In total, 736 reports of functional limitations/diseases were given for the 15 kinds of functional limitations/diseases. In the table, *N* stands for number of persons (out of 555 respondents) reporting the different number of functional limitations/diseases. The symbol n stands for subsamples of respondents in each degree of reduction in functional ability; ^3^ Response categories for functional ability: (a) not reduced; (b) somewhat reduced; (c) reduced; (d) very reduced; (e) extremely reduced. ^4^ Includes respondents with kinds of functional limitations/diseases *additional* to the 15 response alternatives listed in the questionnaire.

#### 4.6.5. Model B—Identify Barriers for Travelers with Different Degrees of Functional Ability

For respondents with a *high functional ability*, “price” and “time-keeping” were the two most important barriers for traveling more often with *long-distance trains* (see Appendix for the complete list of 30 barriers; please note that the question was formulated as potential “barrier” reductions): 62% (or 292 persons) said they would travel more often if it became less expensive to travel. Price was an important barrier also for persons with low functional ability (41% or nine respondents). In the higher functional-ability group, 50% (or 226 persons) would travel more often if departure and arrival times were kept (23% or five persons in the low functional-ability group, Cramér’s *V* = 0.16, *p* = 0.043). However, for the respondents with “low/extremely low” (50% or 11 respondents) compared to those with “high” functional ability (19% or 88 respondents), the most important prerequisite for traveling more often was regarded “to be healthier” (Cramér’s *V* = 0.16, *p* = 0.002). This association was not secured for any of the *specific* functional limitations/diseases here researched, see Section 4.6.1.

#### 4.6.6. Model B—Travel Behavior as a Function of Travelers’ Degrees of Functional Ability

**Figure 3:**
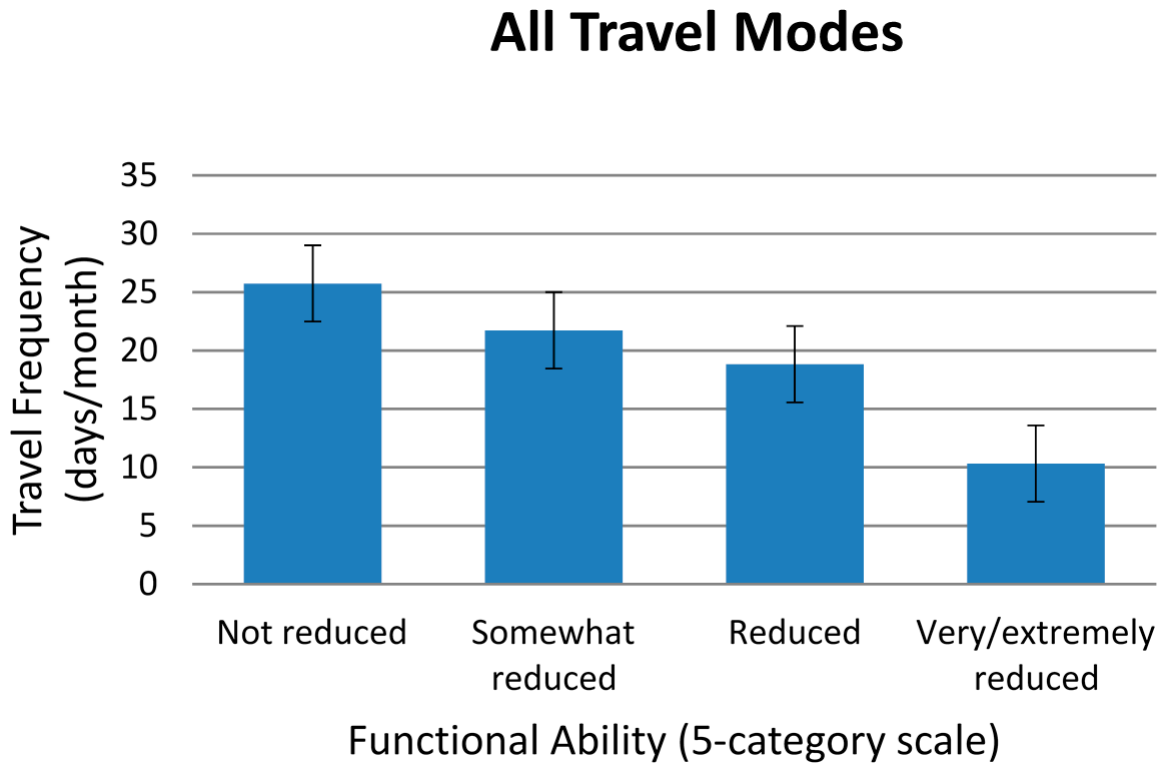
Travel behavior as a function of functional ability for all travel modes combined: Mean travel frequency in days per month traveled, presented as a function of the respondents’ reduction in functional ability. The travel modes were: long-distance and short-distance train, bus, airplane, boat, and car, taxi inclusive, as well as mobility service.

**Figure 4:**
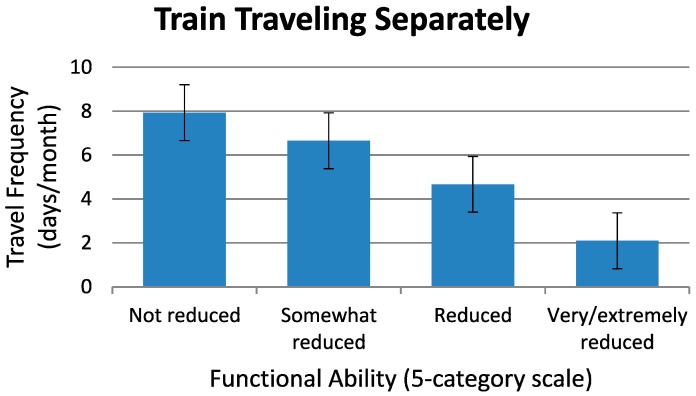
Travel behavior as a function of functional ability for travels by rail: Mean travel frequency in days per month involving various kinds of rail bound modes (long-distance train, commuter train, the underground and local train), presented as a function of functional ability.

Figure 3 shows the main result on traveling with all travel modes combined (long- and short-distance trains, bus, airplane, boat, and car, taxi inclusive, as well as mobility service). That is, with travelers’ improved functional ability, the average travel frequency per month will increase. This result indicates that the *lower* the functional ability of the potential travelers, the *higher* the probability that their travel frequency would go down. The same pattern of results is found to be true for train traveling separately, Figure 4. In both Figure 3 and Figure 4, the ordinate shows the mean travel frequency per month with its 95% confidence interval indicated.

Respondents *with* the lowest degrees of functional ability (“very reduced” and “extremely reduced”) traveled 10 days per month with all travel modes combined *or* 2 days per month by train only. These two travel frequencies should be compared to 26 *or* 8 days per month, respectively, for the respondents *without* reduced functional ability. Accordingly, respondents who considered their functional ability to be high had more often taken *long-distance train* trips last year than those who reported that their functional ability was low. Among the respondents *with* very or extremely reduced functional ability, only 9% had the last year been traveling with long-distance trains, as compared to 31% in the group *without* reduced ability. For those with reduced functional ability, it was also more common to have traveled the last year by long-distance train if they had a car available in the household (odds ratio 2.2). Income level was not a significant predictor of long-distance train traveling for this group of elderly. The reason for this could be that long-distance traveling by car is more straining than long-distance traveling by train, and that respondents with car in the household might to a greater extent have a travel companion (spouse), thus making train traveling more available.

Regardless of functional ability, the most common purpose of the *latest journey* was “to visit relatives or friends” for the respondents who had traveled by long-distance train the last year (64% for those *without* reduction and 56% for those *with* reduced ability). The second most common purpose of the latest journey for those *without* reduced ability was “entertainment, culture and sport, and other events” (21%) whereas it was “tourist trip” (21%) for those *with* reduced ability. The third most common answer for those *without* reduction was “work or studies” (16%), while those *with* reduction answered “entertainment, culture, sport” (13%).

For the group of respondents *with no* reduction in functional ability, the arithmetic mean travel distance of the last journey was 500 km and mean travel time 5 h. For those *with* reduction in functional ability, corresponding means were for travel distance 600 km and for travel time 21 h, but with a median of 4 h (comparable to the group with no reduction in ability). The large difference between arithmetic mean and median travel time for those with reduced ability is caused by a wide range and a skewed distribution.

In most cases, the destination of the latest journey had been within the country, in this case Sweden: 93% for those without and 90% for those with reduced functional ability. The latest journey had also been without heavy luggage (78% for those without and 69% for those with reduced functional ability) and without children under the age of 6 (99% for those without and 100% for those with reduced functional ability). Most respondents traveled alone (59% for those without and 54% for those with reduced functional ability), whereas the second most common way of traveling was with relatives or friends (37% for those *without* and 46% for those *with* reduced functional ability). Notably, only 1% of those *with* reduced functional ability traveled with a companion.

Most persons changed their modes of transport during their latest long-distance train journey (65% of those *with* and 69% of those *without* reduced ability, respectively). The train was the most commonly used connecting mode (59% of those *with* and 44% of those *without* reduced ability, respectively) followed by bus (40% and 41%, respectively). The most frequent way of getting *to* and *from* the railway stations was by train (55% and 62%, respectively). Please note that “train” here included long-distance train, commuter train, underground, and local train/tram.

To conclude, there was a tendency that our elderly respondents *with reduced functional ability* to a lesser extent had traveled to work (or study) on their last journey, but, notably, they had more often made a tourist trip, instead. They had also, to a higher extent, traveled with relatives or friends and with heavy luggage as compared with those without reduced functional ability.

#### 4.6.7. Model A and B—Interrelationship between Travel Behavior and Barriers

Regarding long-distance trains, some of the desired reductions in barriers (30 in all, see Appendix) for traveling more frequently were weakly associated with reported frequency of *train traveling*. There was a tendency that those who had traveled more frequently, would travel even more often if the particular barriers were removed. The barriers most strongly associated with travel frequency were cost (“less expensive”; Spearman’s ρ = −0.22, for *n* = 357) and ticket purchase (“easiness of buying tickets for the whole journey”; ρ = −0.20, for *n* = 300). For both, the association was significant at the 1% level.

If our set of 30 barriers were correlated with travel frequency for *all travel modes combined*, trains inclusive, five of the barriers produced coefficients of rank-order correlation (ρ), significant at the 1% level. The highest association was for “becoming healthier and therefore being able to travel” (ρ = 0.24, *p* = 0.01, *n* = 269). In this case, it was the respondents who traveled more *infrequently*, who said they would like to travel more often, if their health had been better. The other significant rank-order correlations were for the four barriers: “not afraid of being harassed” (ρ = 0.17, *n* = 306), “cost” (ρ = −0.17, *n* = 329), “other passengers’ attitudes become better” (ρ = 0.16, *n* = 256), and “not having to travel underground” (ρ = 0.16, *n* = 308). Thus, in principle, those who traveled infrequently would travel more often if barriers related to “adverse health” and “low security” were reduced, while frequent travelers would travel more frequently if the barrier of “ticket price” was reduced.

The barrier “weather” influenced some of the respondents’ train traveling. We found that those, who reported railway accessibility to be high, were also more influenced by the weather in traveling as compared to those who reported it to be low (ρ = 0.16, *p* = 0.01 level, *n* = 529). Also, those who traveled frequently by train were slightly more influenced by (bad) weather than the more infrequent travelers (ρ = −0.14, significant on the 5% level, *n* = 332). This result refers only to the trains, because there was no or weak associations with the weather if all travel modes were included in the data analysis.

#### 4.6.8. Model B—Railway Accessibility for Travelers with Different Degrees of Functional Ability

**Figure 5:**
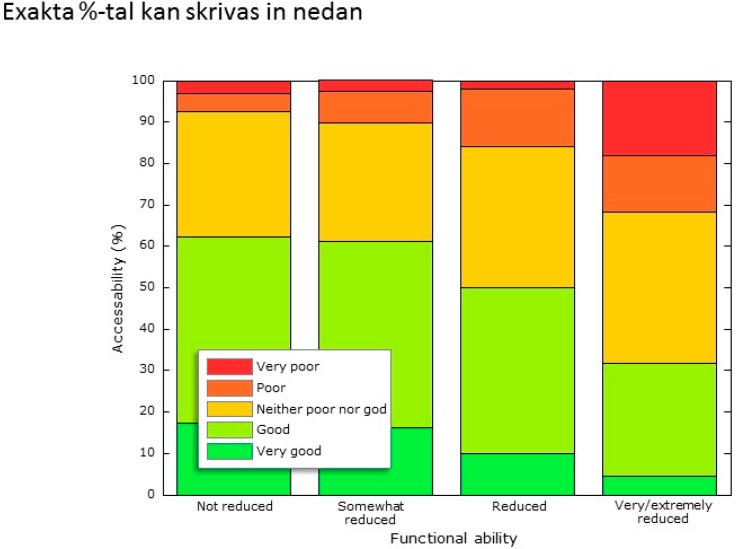
Perceived railway accessibility as a function of functional ability.

Good or very good railway accessibility was reported slightly less frequently by the participants with reduced functional ability (56% out of 219) as compared to those without reduction (62% out of 300), see Figure 5. Total response frequency was 519. None of the (only) three respondents with “extremely reduced” functional ability found the railway accessibility to be good. Logistic regression showed that perceived railway accessibility could not be explained by age, gender, income, or car possession. Among those respondents who had mobility service (*n* = 43), 19 perceived railway accessibility as “neither good nor bad”, whereas six reported it to be “extremely poor” or “rather poor”.

Significant correlations were found between perceived railway accessibility and self-reported degree of functional ability for participants with five of the functional limitations/diseases: neurological disease, chronic pain, restricted mobility, diabetes, and vision impairment. Especially those who reported their functional ability to be low, also scaled railway accessibility as low. Those with neurological diseases had the highest coefficients of rank-order correlation between railway accessibility and degree of functional ability (Spearman’s ρ = 0.85, *n* = 10), also those with chronic pain (ρ = 0.38, *n* = 51) and restricted mobility (ρ = 0.32, *n* = 74) produced significant coefficients of correlation (ρ > 0.30).

Despite the old age of the respondents, a majority of those who “had not been traveling by long-distance train during the last year”, thought that, it might be possible to travel if they would have liked to. Thus, 91% of those without, and 78% of those with reduced functional ability, considered it to be possible for them to travel. Those, who reported it to be impossible were only 2% and 6%, respectively. Logistic regression indicate that respondents who used a walking aid (walker, wheel-chair or crutches) or a hearing aid, were more likely to report railway accessibility as rather poor or extremely poor (odds ratio 3.21 or 2.59, respectively) as compared to those who did not have that kind of aid. Railway accessibility was unrelated to the other aids (e.g., cane, glasses or lenses). Moreover, restricted mobility was correlated, but weakly, with not being able to travel by long-distance train (Spearman’s ρ = 0.26, *p* = 0.01). For *p* = 0.05, significant coefficients of correlation were also found for epilepsy (ρ = 0.13), diabetes (ρ = 0.11), and chronic pain (ρ = 0.10), the total number of respondents on this set of questions is n = 394.

#### 4.6.9. Model A & Model B—Railway Accessibility and Its Relationship to Barriers

Railway accessibility was positively correlated for all the barriers encountered during different parts of the latest long-distance train journey (Spearman’s ρ = 0.30–0.47, all significant at *p* ≤ 0.01, *n* = 123–129). Moreover, railway accessibility was also weakly, but significantly, correlated with the weather (ρ = 0.16, *p* = 0.01, *n* = 529); thus, participants who were influenced by the weather, found the railway system more accessible.

The Spearman’s ρ was generally low, and not significant, between railway accessibility and each of the 30 potential barriers for traveling more often with long-distance train (see list in Appendix). Railway accessibility correlated with the number of barriers reported; the coefficient of correlation was low (ρ = 0.26), but significant at *p* = 0.01, *n* = 140. This result tells us that those potential travelers who perceive railway accessibility to be low, also, to a greater extent would *not* have traveled by train more often, even if the travel environment would be improved. Moreover, wanting to travel more often with train is *not* highly associated with any specific barrier actually encountered. Notably, the problem of “retrieving information at stations” constitutes the long-distance train barrier that has the highest coefficient of correlation with *not* “wanting to travel more often with train” (ρ = 0.30, *p* = 0.01, *n* = 105).

#### 4.6.10. Model A & Model B—Railway Accessibility and Its Relationship to Travel Behavior

**Figure 6:**
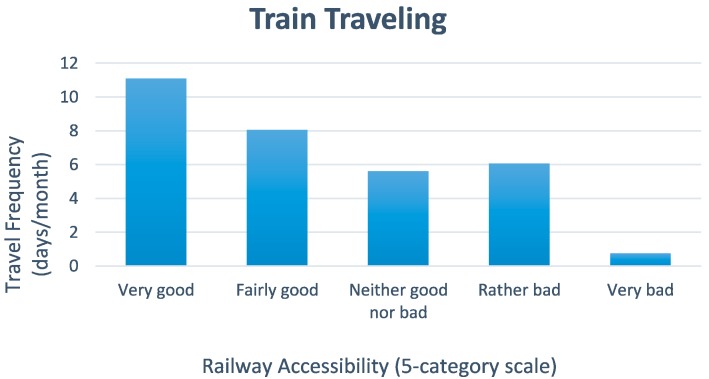
Travel behavior as a function of railway accessibility: Mean travel frequency in days per month traveled by rail involving various kinds of rail bound modes (long-distance train, commuter train, the underground and local train), presented as a function of perceived railway accessibility.

Participants, who reported railway accessibility to be high, were also those that traveled by train more often (Spearman’s ρ = 0.29, *n* = 419) compared to those who reported the railway accessibility to be low (cf. Figure 6). This result was true also for the case when other travel modes were included (ρ = 0.21, *n* = 382). Both these coefficients of correlation are statistically significant at *p* = 0.01. No association was found between how often the respondents traveled (either by train or by all travel modes combined) and whether or not they would have liked to travel by train more often, if it would have been possible. Railway accessibility was weakly associated with choosing the train as the transport mode for journeys abroad (ρ = 0.12, *p* = 0.01, *n* = 528).

#### 4.6.11. Model A & Model B—Railway Accessibility and Its Relationship to Travel Behavior and Barriers for Respondents with Different Kinds of Functional Limitations/Diseases and/or Degrees of Functional Ability

*Functional limitations/diseases (Model A).* Railway accessibility was perceived to be lowest by the respondents who had the two functional limitations of restricted mobility and/or chronic pain. Together with neurological disease, restricted mobility was the functional limitation/disease most highly associated with the barrier “to move around onboard the long-distance train”. Moreover, persons with neurological and/or rheumatic diseases were most troubled by the barrier “moving problems within the stations”. Regarding travel behavior, restricted mobility was again the functional limitation/disease that had the highest coefficient of correlation with travel frequency. Part of these outcomes might be referred to symptoms common to many of the here studied functional limitations/diseases; for example, chronic pain and restricted mobility are both highly associated with neurological disorders, cf. Table 3.

*Functional ability (Model B).* Railway accessibility was perceived to be slightly better for the respondents with higher functional ability. Functional ability is important for the perception of railway accessibility, especially for persons with neurological disease, chronic pain, restricted mobility, diabetes, and vision impairment. For those with a high functional ability, “price” and “time keeping” were reported to be the most important barriers for traveling more often with long-distance trains. Price was important also for persons with low functional ability, but the barrier they most frequently reported was their “own health”. As could be expected, those with high functional ability traveled more frequently than those with low ability. Regarding their most recent journey, travelers with low functional ability had to a lower extent traveled to work (or study) compared to those with high functional ability. On the other hand, those with low ability had more often made a tourist trip, traveled with relatives and friends and traveled with heavy luggage. These results suggest that those with low functional ability travel less frequently but make longer trips by train instead.

## 5. Discussion

The World Health Organization [19] defined disability as the product of the interaction between individual features of a person and individual characteristics of the environment. Disability may refer to anything from inaccessible information, staircases-and-doorsteps to persons’ attitudes (in this research denoted “barriers”). Therefore, the very same functional limitation/disease may become a disability in one environment, but not in another. Moreover, disability and functional limitation/disease should be understood from the perspective of the individual traveler’s personal experiences and interpretations, because these may differ among individuals even if their functional limitations/diseases are of the same kind. In many life situations, it may be fruitful to focus on the individual’s abilities rather than his/her disabilities. After all, it is the individuals’ abilities rather than their disabilities that may help the travelers overcome barriers in the travel environment. It follows that, by identifying and reducing barriers in the travel environment, the functional ability may be improved (cf. Figure 1).

The findings of this research suggest that the respondents’ functional ability, as well as some kinds of functional limitations/diseases, will influence (a) how railway accessibility will be perceived; (b) what barriers will be encountered during door-to-door trips; and (c) how travel behavior will develop. Moreover, the extent to which our elderly respondents found the railway travel environment accessible was associated with how often they traveled. Thus, railway accessibility and travel behavior can be thought of affecting each other. Those who find a travel environment accessible may be inclined to travel more often, but traveling more frequently would also help to overcome potential barriers in the travel chain, such as ticket machines and scarce information. Many of our frequent *train* travelers, often also scoring high in functional ability, reported they would have traveled even more often by long-distance train, primarily, (a) if the ticket price would be reduced and (b) if tickets for the whole trip would be possible to buy at the same occasion. If *all travel means* are included, the respondents’ problem with their *own health* was highest correlated with the wishes to travel more often with long-distance train. In that case, it is the respondents who traveled more infrequently, who said they would travel more often if their own health had been better. Thus, the respondents referred this barrier to themselves. A focus of future research could therefore be on what changes to the transport system might help these potential low-health travelers to overcome essential barriers.

For respondents with some sorts of functional limitations/diseases, the level of their functional ability was found to be associated with how accessible they *perceived* the travel environment to be. The highest coefficients of correlation were obtained for (1) the neurological diseases; (2) chronic pain and (3) restricted mobility. Mild symptoms might not pose any problems in the travel environment, although more severe symptoms of the same functional limitations/diseases might lead to a more challenging travel environment. Seriously restricted mobility (that may be central in all of the three conditions above), might pose problems in the travel environment that other functional limitations/diseases, like hearing impairment, would not. It is thus important to focus on the most affected groups of potential travelers in order to find out, in more detail, what kinds of barriers they would encounter and what might be done to increase accessibility for them. Notably, few of our respondents reported that they were low in functional ability, and this might be a source of uncertainty in our data. Another source of uncertainty is that only few respondents had some of the functional limitations/diseases, e.g., only five persons with epilepsy had scaled railway accessibility, whereas the restricted-mobility group was as large as 74. Moreover, even if there is no direct link between functional ability and railway accessibility for some of the functional-limitation groups, it does not necessarily mean that the respondents do not experience barriers. Even persons without functional limitations would experience certain barriers. For example, during delays, passengers might be equally disturbed, independent of their functional ability.

In this research, pricing was seen as the most important barrier for not traveling more often, indicating that pricing is central to railway accessibility. However, pricing did not correlate with railway accessibility. The reason might be that pricing is not regarded as an accessibility issue. Rather, accessibility might be regarded primarily as a matter of, for instance, the physical environment, information, and/or time keeping.

In cross-sectional study designs, causal relationships cannot be drawn from coefficients of correlations. For causality, a longitudinal design would be necessary, based on repeated measurement of the variables. Moreover, self-reported data should always be interpreted with caution. However, in the present cross-sectional study, direct questions on travel experiences would be less sensitive to various biases (e.g., regarding social desirability), than questions on more complex psychological constructs such as anxiety or depression. Presently, there is a lack of questionnaires targeting accessibility in railway traveling for the elderly. Our exploratory research is intended to form a basis for systematic investigation of overall accessibility as based on interactions among travelers’ functional ability and travel behavior in relation to barriers met during whole-trip traveling; focusing on persons with various kinds of functional limitations.

More than 40% of the respondents said they would like to travel by train more often, indicating an unmet need for older travelers. The overall goal of this paper is to help facilitate the development of a public transport system that would meet the specific needs of vulnerable travelers (and would-be travelers) that are often neglected. Moreover, this exploratory research has improved our knowledge of elderly persons’ perceptions and needs in the public transport system. We are here proposing a prototypical model of measurement that builds on a theoretical model for how accessibility may be understood (Figure 1). We found our five constructs here researched, functional limitation/disease, railway accessibility, functional ability, barriers, and travel behavior, to constitute a useful theoretical basis for a measuring instrument of overall accessibility in the travel environment. Emardson* et al.* [26] created an accessibility measure for persons with functional limitations, based partly on the individual’s perceived effort in facing a certain barrier and partly on the probability of facing such a barrier in traveling. The effort needed to overcome the barrier and to make the journey is grounded in the person’s functional ability. The more weight placed on a certain barrier for an individual, the less probable is the particular journey. In measuring accessibility for vulnerable travelers, it is important that the barriers are representative for travelers with various kinds of functional limitations/diseases. Thus the questionnaire here developed could be used to identify persons, representative for these different groups of travelers.

## 6. Conclusions

In our sample of 574 elderly respondents (65–85 years old), more than a quarter (*n* = 157 or 28%) reported that they had no functional limitations/diseases out of a list of 15. The most common functional limitation was *vision impairment* (*n* = 122 or 22%) followed by *hearing impairment* (*n* = 120 or 21%) and *cardiovascular disease* (*n* = 97 or 17%). Most of our 574 respondents reported that they had a good functional ability (57%), but 5% were found to have severe reductions (thus, 38% had somewhat reduced ability). Severe reduction in functional ability was most frequently reported for two of the functional limitations/diseases, namely “restricted mobility” and “chronic pain” (Table 2). Out of the sample of 555 respondents (cf. Table 5), those with more than one functional limitation/disease reported that their functional ability was lower than the ability of those with “no or only one” functional limitation/disease (35% and 65%, respectively, see Table 5).

On average, our elderly traveled 23 days per month (all travel modes included: long- and short-distance trains, bus, air, boat, and car, taxi inclusive, and mobility service). Car was the most frequently used mode of transport. Travel frequency was found to correlate weakly, but at a statistically significant level, with three out of 15 kinds of functional limitations/diseases, namely, reduced mobility, attention/memory/concentration, and vision impairment. Respondents with their functional ability severely reduced traveled less frequently than those with a higher functional ability.

In travels with long-distance train in the last year, the majority of our elderly respondents found it easy to travel. The most important barriers were experienced at stations and involve inferior/deficient/inconsistent information, problems with orientation, difficulties to move within stations, as well as to get onboard the train, particularly with heavy and bulky luggage. The most important actions that would motivate our elderly respondents to travel more frequently with long-distance train would be to reduce the following two barriers: “expensive tickets” and “too low punctuality”.

### 6.1. Railway Accessibility

Although railway accessibility was regarded to be good by a majority of the elderly participants, 10% reported that they were very discontent. A main question was to research how the elderly’s railway accessibility is related to their functional limitations/diseases, functional ability, travel behavior, and the barriers they have met in their travel environment:

1. *Functional limitations/diseases.* The elderly with the functional limitations/diseases of *restricted mobility* &* chronic pain*, perceived railway accessibility to be lower than those with other types of functional limitations/diseases. For each of five of the functional limitations/diseases (out of 15), *neurological disease, chronic pain, restricted mobility, diabetes* &* vision impairment*, perceived railway accessibility was correlated with the degree of functional ability.
2. *Functional ability.* For all elderly participants taken together those who had a high functional ability (particularly participants with travel sickness, diabetes and asthma/allergy/hypersensitivity) also found the railway accessibility (*n* = 519), to be higher than those who had a low functional ability.
3. *Barriers.* For the most recent long-distance train journey, railway accessibility was weakly correlated with all barriers that had been encountered during the whole trip, the *weather* inclusive. Those who perceived railway accessibility to be low, also to a greater extent reported that they would not travel by train more often, even if barriers were removed and the travel environment would be improved.
4. *Travel behavior.* The elderly who perceived railway accessibility to be high, also reported that they traveled more often (travel behavior) than those who perceived it to be low. That is, the better the railway accessibility, the higher the travel frequency.

### 6.2. Overall Accessibility

As illustrated in Figure 1, overall accessibility is modeled from respondents’ various kinds of functional limitations/diseases (Model A), or their levels of functional ability (Model B), together with their travel behaviors, and the barriers they encounter during trips in the travel environment. In the questionnaire, *overall accessibility* has been measured with the aid of one question on *railway accessibility*. All our five target constructs were found to be associated in a meaningful way and thus together create the overarching concept of overall accessibility. The constructs researched were: functional limitation/disease, functional ability, barriers, travel behavior, and railway accessibility.

### 6.3. Barriers to Be Remedied for a More Independent Travel Behavior

A main research goal was to determine, in the whole-trip travel environment, what *barriers* are encountered by the elderly with varying functional ability and potential functional limitations/diseases. Another goal was to determine the elderly’s travel behavior interactively with the barriers they encounter in whole-trip traveling. Finally, we wanted to find out what are the most important barriers these older persons would encounter in train traveling? We found the following:

1. Different kinds of barriers dominate among the travelers with different kinds of functional limitations/diseases. For example, persons with neurological and rheumatic diseases and those with restricted mobility, found it “difficult to move around onboard long-distance trains” or “move around within stations”. No other associations were established between specific barriers and the kinds of functional limitations/diseases of our elderly participants.
2. “Lower ticket price” and “shorter travel time/better punctuality” would help to reduce/remove barriers that are central to the elderly travelers with high functional ability.
3. Compared to our other respondents, the elderly with the most severely reduced functional ability more often attributed barriers to their “own health”.
4. For elderly who are already frequent train travelers, “lower ticket prices” and “ease of buying tickets for the whole journey” were important barriers to more frequent train traveling.

Practice and policy implications for service providers would include improved personal information combined with way-finding services and repeated visual and auditory information along the transition routes. Moreover, in the long term, design improvements, particularly step-free access, would reduce important barriers.

## Appendix

**Table A1:** Thirty potential barriers for traveling by long-distance train more often.

Question:	Response
I Would Travel More Often by Train, If…	Frequency *
If it would be less expensive to travel	62%
If departure and arrival times were kept	49%
If I knew I would be in time at the final destination	48%
If I knew I would be in time for my connection	47%
If I would not have to change modes of travel during the trip	43%
If I knew I would get help if I need	42%
If it would not be crowded onboard	40%
If I knew I would be in time for the long-distance train	33%
If the travel time would become shorter	33%
If it would be easier to find an empty seat	33%
If it would be easier to book/purchase tickets for the whole trip at the same time (even connections)	33%
If there were service staff at the platform	29%
If I would feel secure going to and from the station	26%
If the attitude of the staff would be more service minded	26%
If I would not be afraid of being harassed	25%
If I could be sure I would manage the whole journey	25%
If it would become easier to get help from staff onboard	25%
If it would become easier to book/purchase tickets	24%
If the environment would become less busy	24%
If I would have more time to get on or off the train	24%
If the departures were more frequent	22%
If it were easier to get help from staff within the station area	22%
If I were healthier and therefore could manage to travel	20%
If the staff would be more proficient	20%
If it would be easier to park at the station	19%
If I would not have to keep track of so many things during the trip	18%
If trains and stations were designed in a more homogenous way	15%
If it would be possible to travel at other hours (for example at night)	11%
If fellow passengers’ attitudes were better	11%
If I would not have to travel under the ground	9%

***** Depending on question, the response frequency is *n* = 483–506.
